# The organization of verb meaning in Lengua de Señas Nicaragüense (LSN): Sequential or simultaneous structures?

**DOI:** 10.16995/glossa.10342

**Published:** 2024-02-09

**Authors:** Diane Brentari, Susan Goldin-Meadow, Laura Horton, Ann Senghas, Marie Coppola

**Affiliations:** University of Chicago, US; University of Chicago, US; University of Wisconsin-Madison, US; Barnard College, Columbia University, US; University of Connecticut, US

## Abstract

One structural dimension that varies across languages is the simultaneous or sequential expression of meaning. Complex predicates can layer meanings together simultaneously in a single-verb predicate (SVP) or distribute them sequentially in a multiple-verb predicate (MVP). We ask whether typological variability in this dimension might be a consequence of systematic patterns of diachronic change. We examine the distribution of markers of agency and number within the verb phrase (the predicate) in the earliest stages of a young, emerging sign language in Nicaragua, Lengua de Señas Nicaragüense (LSN), beginning with homesign systems like those from which LSN originated, and progressing through two decades of transmission to new learners. We find that: (i) LSN2 signers are more likely to produce MVPs than homesigners or LSN1 signers; (ii) in the MVPs they do produce, homesigners and LSN1 signers are more likely to produce predicates that mark both agency and number *simultaneously* on at least one of the verbs; LSN2 signers are just as likely to produce sequences with verbs that mark agency and number in *sequentially* separate verbs. We discuss how language acquisition, modality, and structure, as well as specific social factors associated with each of the groups, play a role in driving these changes, and how, over time, these patterns of change might yield the diversity of forms observed across spoken and signed languages today.

## 1 Introduction

A persistent puzzle in every domain of linguistics is typological variability: why do present-day languages encode and organize similar information in different ways? The answer to this question likely lies in the dynamic diachronic changes in each language’s history as it was transmitted and used over generations (Weinreich 1953; Hock & Joseph 1996; Hurford 2012) and affected by cycles of change due to factors associated with variability in the language ecology (Haugen 1972; Ellis & Larson-Freeman 2006; Hudson 2019). One structural dimension that varies across languages is the simultaneous or sequential expression of meaningful elements. This dimension also varies within individual languages, with shifts from one of these types of information packaging to the other over time (Bowern et al. 2008; Aikhenvald 2011; 2018). Linguistic structures may be holistic, containing no componentiality, or they may be segmentable into independent components, produced simultaneously or in a sequence. In the current study, we have an opportunity to observe this kind of language variation and change in its most dynamic state – in the earliest moments of the creation of Lengua de Señas Nicaragüense (LSN, also known as Nicaraguan Sign Language), which is approximately 50 years old. We ask whether similar patterns of change are found across signed and spoken languages, and what determines whether diverse form-meaning mappings are grouped together or split apart. Although this topic overlaps with broader issues of lexicalization, the main goal of this paper is to address variation in simultaneous and sequential forms under two conditions—when the form-meaning pairs are stable, and when the signers have the option of sequential, multiple-verb predicates instead of, or in addition to, simultaneously organized, morphologically complex single-verb predicates.

We focus specifically on combinations of meanings associated with concepts of agency (agent/no-agent) and number (singular/plural) in single-verb predicates (SVPs) and in multiple-verb predicates (MVPs). We chose these forms because the form-meaning pairings for agency and number appear to be extremely stable even as the SVP or MVP structure varies. Two of the most studied types of MVPs across both spoken and sign languages are serial verb constructions and resultatives. Serial verb constructions have been documented in Sign Language of the Netherlands (NGT; Bos 2016; Couvee & Pfau 2018) and Hong Kong Sign Language (HKSL: Lau 2012), and resultative MVPs have been found in German Sign Language (DGS) and American Sign Language (ASL) (Loos 2017). The verb sequences under investigation here include sequences that show characteristics of serial verbs and resultatives, as well as other combinations of verbs that make up the broader class of multiple-verb predicates (MVPs). We are examining the broader issue of simultaneity and sequentiality of form and do not focus on any one type of MVP here.

Extensive comparative studies of variation and change in SVPs and MVPs have been carried out in spoken languages, and there is a growing body of work in sign languages as well. Representative works on the topic of the historical change from MVPs to SVPs in spoken languages include Lefebvre (1991), Bowern (2006), Bowern et al. (2008), Amberber et al. (2010), Aikhenvald & Muysken (2011), and Aikhenvald (2018). Aikhenvald (2018) describes the rise and fall of MVPs in spoken languages, historically speaking. With respect to their origin (the “rise” of MVPs), all MVPs originate from single words (free morphemes) whose sequence has become conventionalized over time to form two types of MVPs: asymmetrical (examples 1–2) or symmetrical (example 3). At later stages of historical change, asymmetrical and symmetrical MVPs tend to undergo different paths towards simultaneity in lexicalization (the “fall” of MVPs). In *asymmetrical* MVPs, where one of the two verbs carries all of the morphology and the other carries none, the ‘light’ or ‘minor’ verb can become grammaticalized as an affix or clitic. An asymmetrical MVP is given in (1) from Yurakaré (van Gijn 2011); *poyde* is the light verb and has no morphology. In Edo the root /**rē**/ (Eng: *come*), occurs in the language as an independent verb (2a), as a light verb (2b), and as an affix (2c; Agheyisi 1986; Lord 1993). In *symmetrical* MVPs, where both verbs allow morphological affixation, the two verbs become a conventionalized lexical combination, sometimes with a new, non-compositional meaning. A symmetrical MVP is given in (3) from Watam (Foley 2008); the realis suffix -*r/ur* appears on all of the verbs *uŋg* (Eng.: *pull*), *irki* (Eng.: *go-down*), and *mamai* (Eng.: *finish*). In both asymmetrical and symmetrical MVPs, several meanings are incorporated within a single verb over time.

1. Asymmetrical MVP that contains the meaning “causative” (from Yurakaré, a language isolate of Bolivia, van Gijn 2011:269)
  *nij* V1:***poyde*** V2: ***ka_dyomoj_che_y*** *ëshshë*
  neg **be-able 3sg_go-up_caus_1sg.s/a** stone
  ‘I could not lift that stone.’
2. Asymmetical MVP that contains the verb “come” as a (a) main verb, (b) light verb, and (c) affix (from Edo, a Kwa language of Nigeria; Lord, 1993:231–23, originally from Agheyisi, 1986)
  - ***rɛ*** as main verb, meaning *come*
    *òzó* V1:***rē*** *owà $v\overset{\prime }{\tilde{\varepsilon }}$*
    Ozo **came** house my
    ‘Ozo came to my house.’
  - ***rɛ*** as light verb in asymmetrical serial verb construction
    *òzó V1:****voxó*** $èř\overset{\prime }{\tilde{a}}$ *V2:* ***rē***
    Ozo **bent** tree **come**
    ‘Ozo bent the tree over.’
  - as an affix meaning
    *òzó V1:$řy\bar{ɔ}-\underline{\text{re}}$ nέ*
    Ozo woke**-****(up)** already
    ‘Ozo has already woken up.’
3. Symmetrical MVP contains the meaning “realis” (Watam, a Ramu language of Papua New Guinea, Foley 2008:100)
  *yak kor V1:uŋg-****ur*** *V2: irki-****r*** *V3:mamai-****r***
  1sg canoe pull-**realis** go.down-**realis** finish- **realis**
  ‘I finished pulling the canoe into the water’

Lexicalization in signed languages also displays change toward simultaneous forms – for example, in compounding (Liddell & Johnson 1986) and fingerspelled forms (Battison 1978); see Brentari and Padden (2001), Lepic (2019) and van der Kooij et al. (2023) for discussions of the wider issue of lexicalization processes in sign languages. In any case, although an analysis of a single language’s trajectory during language change can add to our knowledge of what is possible in languages, it cannot provide evidence for a universal preference for either simultaneous or sequential morphological packaging in predicates.

The reasons why language change might not follow a universal trajectory are multi-faceted. First, language change is not linear, and is more likely cyclic (Joseph & Janda 1988). Second, language change is subject to dynamic social, cultural, and ecological factors. In a spoken language, if we consider Labov’s 1963 classic study of the phoneme /r/ on Martha’s Vineyard, for example, we see a wide range of social factors that might have influenced change over time. The same forces can be seen in the dynamic cycles of language use, language change, language perception, and language learning in the interactions of members of language communities (Ellis & Larson-Freeman 2006). In Tariana (4), for example, we see a new type of MVP arising as a result of contact with Tukano, the main contact language for Tariana. Tukano is used on a day-to-day basis by most extant speakers. Tukano employs a serial verb strategy to express reciprocal meanings (Aikhenvald 2003; 2022), and this has spread to Tariana.

- New reciprocal MVP in Tariana, Maipurean language of Brazil (Aikhenvald 2022:223)
  *Yawi-nhe* V1:***na****-sape-pidana* V2: ***na****-siwa-kaka diha-dapana-se*
  Yawi-foc **3pl**_speak **3 pl_**do_together art-masc.sg._cl house-loc
  ‘The Yawi (jaguars) were talking together to each other in that (house).’

Third, languages exploit a wide range of possibilities to express phenomena such as transitivity, number relations, animacy, etc. The particular morphemes, words, or particles found in a language at a given moment will have an effect on how change takes place and is expressed. In addition, many factors about the process of language acquisition shape the way historical change unfolds (see Senghas 1995; Lightfoot 2010, 2020). Thus, each language handles variation and change within the system of structures and contrasts available to it, and the language accommodates the change, with cascading effects, as it is transmitted to a new generation. Finally, modality (signed vs. spoken) can also be an important contributing factor. These factors give rise to a recurring cycle from SVP to MVP and back to SVP. We expand on these points in Section 5.

The task of reconstructing language change is made more difficult because lexicalization often makes the original phonological form of a word or morpheme inaccessible to future generations. To circumvent this problem, here we analyze meanings whose forms retain the same phonological shape in SVPs and in MVPs—in other words, the same forms are either layered onto a single verb or distributed across multiple verbs.

Examples (1)–(4) show how spoken languages lexicalize and grammaticalize MVPs over time, with a tendency toward SVPs in historical change (Aikhenvald, 2011). In the current study, we ask what happens in the very first steps as a sign language is born. The main comparison addressed concerns “word” vs. “word+word” combinations in complex predicates across three groups of Nicaraguan signers. We ask whether complex predicates are produced more frequently as SVPs (single signs) or MVPs (multiple signs).

## 2 Background

In this section we provide background on several dimensions of the current investigation. First, we describe the nature of simultaneity and sequentiality in sign languages in a general way (Section 2.1). Next, we introduce previous work on single-verb predicates (SVPs) and multiple-verb predicates (MVPs) crosslinguistically and in LSN (Section 2.2). We then provide essential background on the Nicaraguan deaf community (Section 2.3), and we conclude with the specific research questions we pose in the current study (Section 2.4).

### 2.1 Simultaneity and sequentiality in sign languages

A large body of research on sign languages has documented how form-meaning pairings are packaged into words using more simultaneous layering in signed languages than in spoken languages (Meier 1993; 2002; 2012; Brentari 2002; Aronoff et al. 2005; Morgan 2014). An explanation frequently proposed for this tendency is *modality*: a simultaneous signal may be easier to parse when the co-occurring morphemes are processed by the visual rather than auditory modality (Meier 2002; 2012 and references therein). However, constraints on simultaneity can also be shaped by phonology, semantics, cognition, and linguistic structure. For excellent current summaries of simultaneity and sequentiality in sign languages see Vermeerbergen et al. (2007) and Loos et al. (2022).

**Figure 1:**
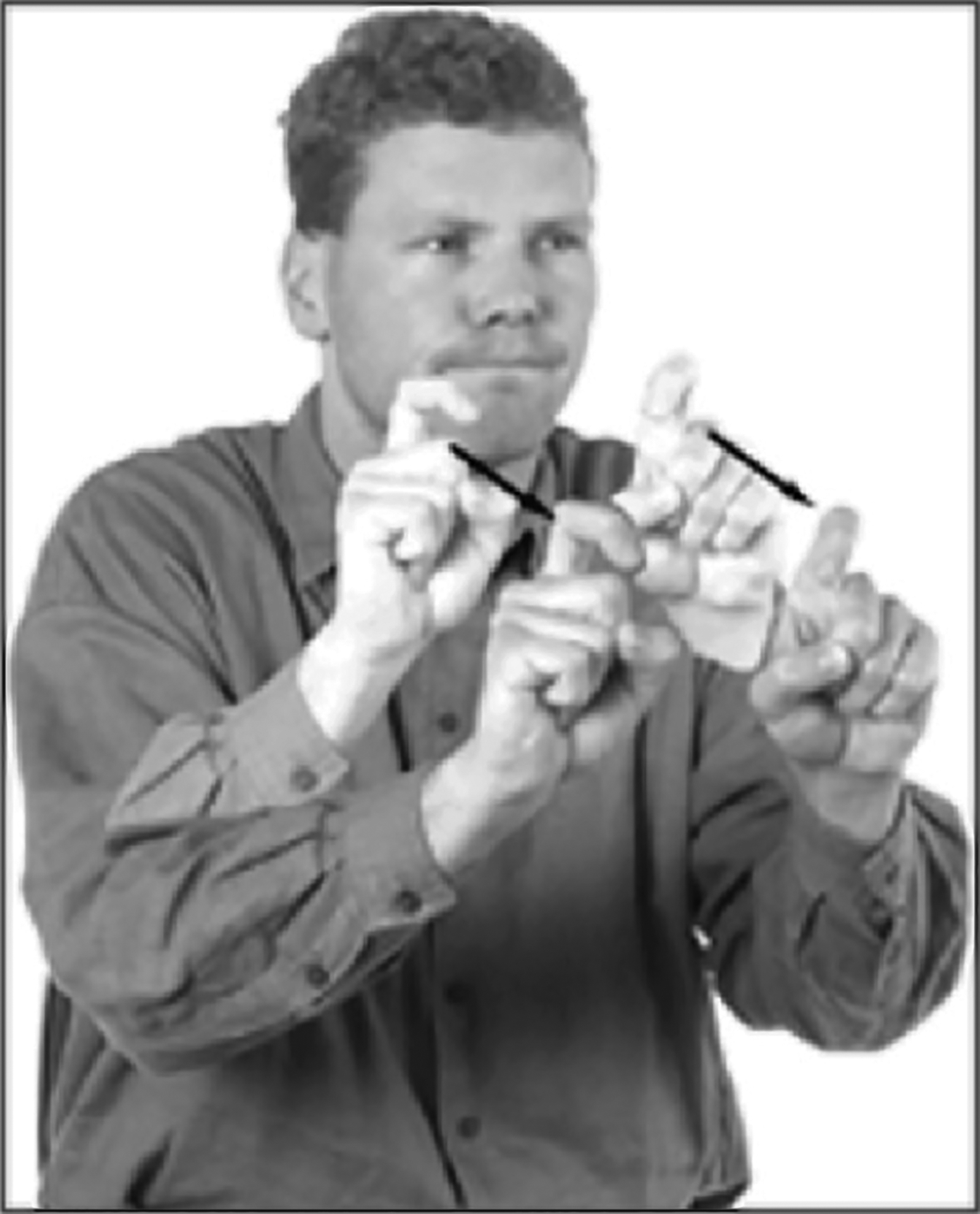
A predicate structure from American Sign Language (ASL) that means two-frail-humans+move- forward+carefully+side-by-side containing five meaningful simultaneous components—’two’ is represented by the hands, ‘people’ by the two index fingers, ‘frail’ by the two bent knuckles, ‘move-forward’ by the direction of movement away from the signer, and ‘carefully’ by the pressed lips. [Reprinted with permission from Brentari, 1998].

Multiple independent manual structures (e.g., movement, location, handshape) can be produced simultaneously, and indeed, signers and silent gesturers have been found to produce high levels of simultaneously layered information to express complex events (Meier 1993; 2002; 2012; Goldin-Meadow et al. 1996; Brentari 2002; Aronoff et al. 2005; Morgan 2014; Slonimska et al. 2020; 2022). This suggests that the visual/manual channel facilitates the use of simultaneous structures. This type of simultaneity is not as likely in spoken language, in part because articulators in the oral modality are less independent than articulators in the manual modality (Brentari 1998; 2002; 2019; van der Hulst & van der Kooij 2005). Silent gesturers have been found to combine up to 5 different movement components within a single complex gesture; however, on average, signers combine more components, use redundancy more frequently in those simultaneous structures, and are more adept at coordinating simultaneous components within a single expression than silent gesturers (Slonimska et al. 2020; 2022). In Figure 1, we see a structure with five simultaneous structures from American Sign Language that means two-frail-humans move-forward-carefully-side-by side: the meaning ‘two’ is represented by the two hands, ‘people’ by the index fingers, ‘frail’ by the two bent knuckles, ‘move-forward’ by the direction of movement away from the signer, and ‘carefully’ by the pressed lips.

Sign language morphology can also appear sequentially, however, and a sign language may tend toward sequential structures as it undergoes certain changes. There are numerous types of sequential structures in sign languages, and we provide a few examples here. Fischer and Janis (1990) analyzed sequential morphology in ‘verb sandwiches’ in ASL, separating a bare form of a verb from the a copy of the same verb with aspectual morphology in a single clause; see (5) from Irish Sign Language (Leeson 1996). Aronoff et al. (2005) suggest that simultaneous morphology is the initial state in an emerging sign language, and that sequential morphology requires time to emerge. Results from the current study suggest that time is not the only factor.

- Sequential Irish Sign Language “verb sandwich” structure (ISL, cf. Leeson 1996:31)
  *my friend both-of us* V1: ***run*** *pant* V2: ***run-with-difficulty***
  my friend dual **run** pant **run-effortful**
  ‘My friend and I were running with difficulty.’

Some two-handed reciprocal forms in German Sign Language (DGS) are also sequential (Pfau & Steinbach 2003(6)). The verb direction moves from the first argument to the second in V1, and then from the second argument back to the first in V2; in this version of the sign the two hands move together in the same direction at the same time.

- Sequential reciprocal two-handed structure in German Sign Language (DGS, Pfau & Steinbach 2003:29)
  *we both* V1:_***1***_***help***_***2***_ V2: _***2***_***help***_***1***_
  1pl both **signerSubj_1sg_ help_(Obj) 2sg (Subj)2sg_help_signerObj_1sg**
  ‘We help each other.’

As a third example of sequential structure in sign language, (7) presents an example of a resultative structure in Hong Kong Sign Language (HKSL, Lau 2012) involving the spread of both dominant hand (H1) and the non-dominant hand (H2)—that is, both hands are held in place across subsequent signs.

- Sequential resultative 2-handed structure in Hong Kong Sign Language (HKSL, cf. Lau 2012:205)
  H1 *sun V1:* ***shine-on****________________________*
  H2 *ice cream___________________________ V2:****melt***
  sun ice cream **shine-on** **melt**
  ‘The sun shone on the ice cream and it melted.’

Even events that occur simultaneously can be pulled apart and represented sequentially within a predicate, as shown in (5) and below in (8). In British Sign Language (Morgan et al. 2002), we see sequential structures representing two perspectives. Some 2-participant events require the signer to locate the two referents in signing space through spatial indexing, and then articulate the main verb (V) from two shifting perspectives. V1 is produced with the perspective of the signer as the subject, and V2 is produced with the signer as the object. These structures are often used when depicting an action that takes place on a specific body part.

- Sequential BSL structure from two perspectives (BSL, Morgan et al. 2002:662)
  *girl*_*j*_ *boy*_*k*_ V1: _***j***_***hit***_***k***_ V2: _***k***_***hit***_***i***_
  girl-locus_j_ boy-locus_k_ **subj_hit_ obj subj get-hit-by_obj**
  ‘The girl hit the boy in the face.’

Forms such as these are also observed in LSN (Senghas et al. 1997; Flaherty 2014). Senghas et al. (1997) found that, when both the grammatical subject and object are animate, an MVP is often required in the clause and SVPs are rare. Senghas et al. (1997) has also documented a change in word order of the MVPs over LSN’s first two decades of emergence, from an NVNV structure (e.g., man push woman be-pushed), to an NNVV structure (e.g., man woman push be-pushed).^1^

Note that there are different types of redundancy in each of the examples (5)–(8); The verb stems are the same in V1 and V2 in (5), (6) and (8), and in (7) the articulation of the V1 is held across V2 so that both hands are in the signing space at the same time.

In the current study, we examine variation in the sign vs. sign+sign structures associated with meanings of agency (agent/no-agent) and number (singular/plural) in iconic signs that are found in most known sign languages––classifier constructions (sometimes referred to as “depicting constructions”; see Supalla 1982; Zwitserlood 2012, and references therein). We chose these forms because they have been observed in homesign (Coppola et al. 2013) and LSN (Goldin-Meadow et al. 2015a), and because we could document them even in the absence of a stable lexicon or “core” vocabulary in homesigners (Richie et al. 2014). The meanings in a classifier construction are conveyed iconically by the individual parameters of the sign. Each parameter in a classifier construction—handshape, movement, location, orientation, and non-manual behaviors—can convey a discrete meaning, which is productively combined with the meanings of the other parameters. Parameters thus function as individual morphemes with movement functioning as a ‘light’ verbal root (either move or be-located), and handshape functioning as a classifier affix (Supalla 1982; Kegl 1990; Schick 1990; Brentari & Padden 2001; Emmorey 2003; Zwitserlood 2003; 2012; Benedicto & Brentari 2004). As noted earlier, the phonological shape of a specific form-meaning pairing in handshape, movement or location is the same whether it is produced simultaneously or sequentially with other forms. The difference lies in their temporal organization.

**Figure 2:**
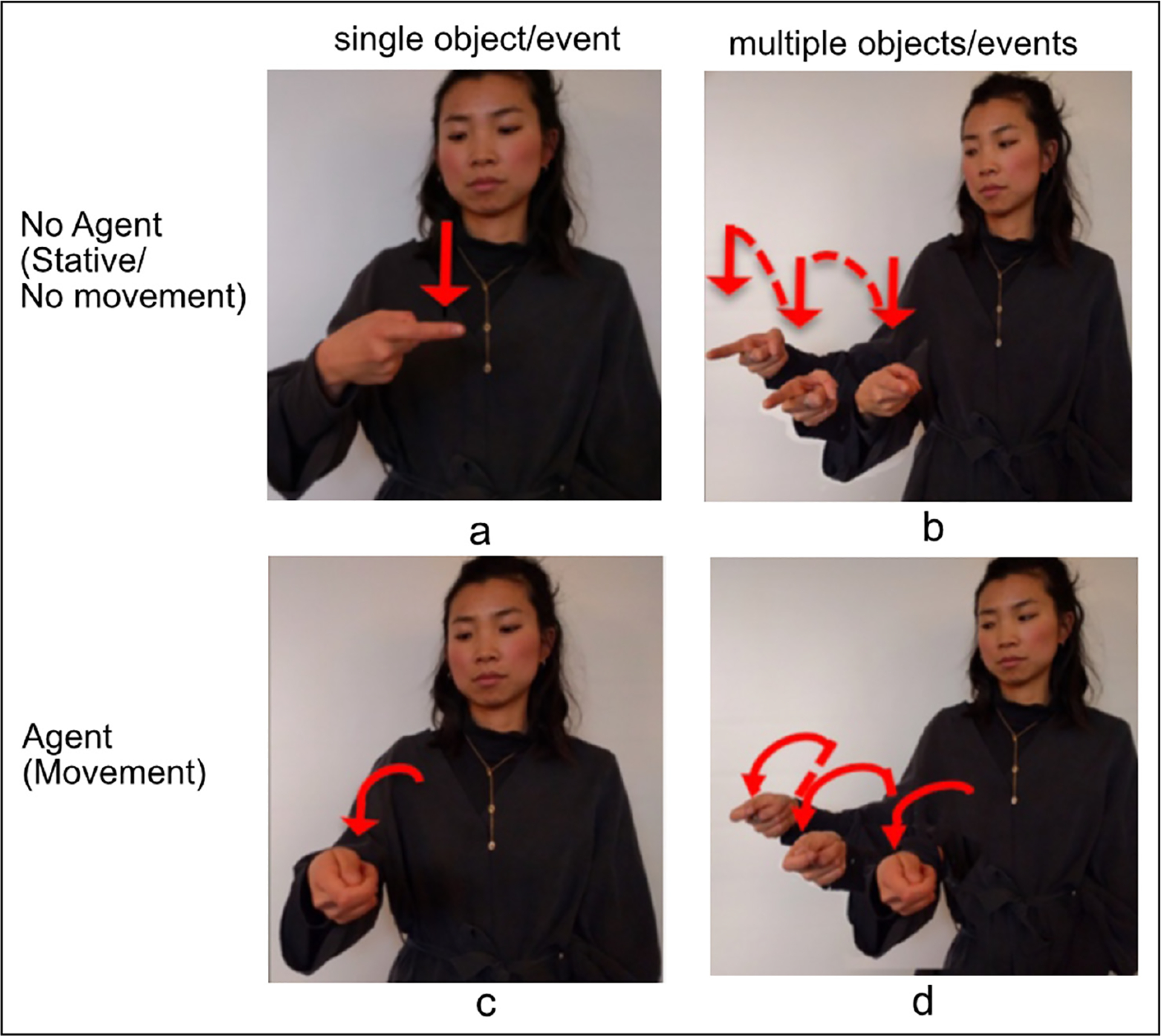
Still images drawn from classifier constructions in ASL with the following glosses in English (descriptions of the scene described follow each gloss): (a) **Pen**-on-horizontal-surface (scene: no-agent/single object/no movement); (b) **Pens****-**on-horizontal-surface (scene: no-agent/multiple objects/no movement); (c) **Someone**-put-pen-on-horizontal-surface (scene: agent/single event/movement); (d) **Someone**-put-pens-on-horizontal-surface (scene: agent/multiple event/movement) [Images reprinted from Brentari et al. 2020 with permission].

**Figure 3:**
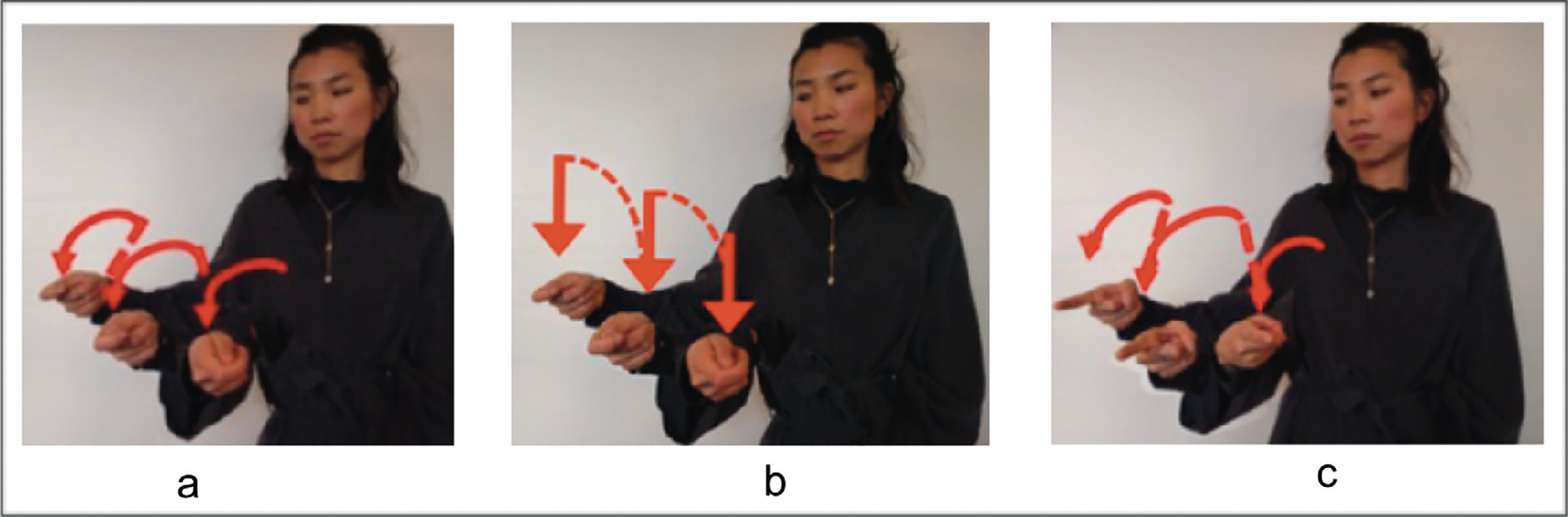
Single verb predicates (SVPs), repeated to express plurality, illustrating: (a) redundant marking for agent on the handshape (handlingHS) and movement axis (midsagittal); (b) agent marking on handshape only (handlingHS); (c) agent marking on the movement axis only (midsagittal).

In classifiers, *agency* is associated with both the handshape parameter and movement axis, and *number* is associated with movement repetition. In order to introduce the relevant forms, Figure 2 presents SVP examples of classifier predicates illustrating the handshape types and movement properties that are analyzed in this work. Combinations involving MVPs are illustrated in Figure 3.

Figure 2 gives examples of a *no-agent* meaning, ***pen****-on-horizontal-surface* (Figure 2a, 2b, intransitive/stative/no-agent) and a contrasting *agent* meaning ***Someone****-put-pen-on-horizontal-surface* (Figure 2c, 2d transitive/agent). The figure also gives examples of a *singular* meaning, ***pen****-on-horizontal-surface* (Figure 2a, 2c; singular) and a contrasting *plural* meaning, ***pens******-****on-horizontal-surface* (Figure 2b, 2d; plural). The following three phonological properties that express agency and number in these types of sign language classifier predicates will be analyzed.

#### Agency/Handshape.

Across sign languages, *object* handshapes are used to express intransitive, non-agentive clauses, and *handling* handshapes are used to express transitive, agentive clauses (Zwitserlood 2003; Benedicto & Brentari 2004; Brentari et al. 2012; 2015a; 2015b; 2016; Mazzoni 2008; Goldin-Meadow et al. 2015a; Rissman et al. 2020).^2^ The two classes of handshape are defined by their iconic properties. Object handshapes use hand-as-object iconicity, and handling handshapes use hand-as-hand iconicity.^3^ Accordingly, Figures 2a and 2b show an object handshape representing a long, thin object in a location without an agent; Figures 2c and 2d show a handling handshape representing how a long, thin object is moved by an agent.

Using the hand to represent the hand when there is an agent present and using the hand to represent the object when an agent is not present may seem like an obvious choice. However, hearing speakers do not produce this distinctive pattern when asked to describe these types of events using gesture without speech. Gesturers readily produce handling handshapes, which use hand-as-hand iconicity, but they produce few object handshapes in the non-agentive predicate context (Brentari et al. 2012; 2015a; 2016).^4^

#### Agency/Movement axis.

In Figures 2a and 2b, the signer produces a downward movement in neutral space without reference to the body. This is a **lateral** axis of movement, in this case a straight vertical movement (in the y-plane) at a perpendicular angle to a horizonal plane in front of the signer with no reference to the signer in the movement itself. Lateral movements are associated with a no-agent, intransitive meaning. In Figures 2c and 2d, the signer produces a movement anchored at the signer’s body, which then moves away from the body. This is a **midsagittal** axis of movement (z-axis, toward/away from the signer), which is associated with an agentive, transitive meaning. In this way, the movement axis, along with *object* and *handling* handshapes, is used to mark the agent/no-agent distinction (Horton et al. 2015). In Figure 2, we see agency expressed redundantly on the handshape and movement axis, but it is possible for the handshape and movement axes to represent different meanings (e.g., a lateral [no-agent] axis combined with a handling [agent] handshape).

#### Movement repetition.

In Figures 2a and 2c the signer produces one movement with a single trajectory. In Figures 2b and 2d, the signer produces repetitions of the movement trajectory. Prior crosslinguistic work on number marking in sign languages has shown that movement repetition often iconically maps onto objects or multiple events—also referred to as pluractional verbs (Kuhn & Aristodemo 2017; Kuhn 2019)—both in classifier constructions (which express spatial events, e.g., Coppola et al. 2013) and in other vocabulary. Expressing multiple nouns or verbs via repetition can be restricted depending on the base lexical item (Fischer 1973; Fischer & Gough 1978; Zwitserlood & Nijhoff 1999; Pfau & Steinbach 2003; 2005; 2006; Hou 2013; Börstell et al. 2016; van Boven 2021). We focus on plural forms marked with movement repetition in the light verbs move and be-located; in such cases the repetition can also include information about the spatial arrangement of the objects under discussion (Pfau & Steinbach 2005)

### 2.2 Previous work on variation in sequential and simultaneous structures in predicates

Studies on LSN (Senghas et al. 2004, 2013) and other sign languages (Brentari et al. 2020) lay the groundwork for the current study of diachronic variation in an emerging language. Senghas et al. (2004) found that one group of LSN signers was more likely to split manner and path into two signs—V1 and V2, as in (9b)—than Spanish-speakers, who tended to combine manner and path simultaneously in a single conflated gesture produced along with speech (9a). This type of sequential structure is also documented in ASL (Supalla 1990). The iconicity of the time of the event is lost since both manner and path occur at the same time in the event being described. These results highlight a contrast between gesturers’ (simultaneous) and signers’ (sequential) preferences, showing that the visual-manual modality does not always favor simultaneous over sequential forms (see also Özçalışkan et al. 2016).

- Combinations of manner and path in Nicaraguan Spanish co-speech gestures (a) and LSN2
  - Simultaneous structure of manner and path in Nicaraguan co-speech gesture (Senghas et al. 2004: 1780)
    *[cat]* V1: *move_****roll +downhill***
    [cat] move_**Manner_path**
    ‘The cat rolled down the hill.’
  - Sequential structure of manner and path in LSN (Senghas et al. 2004: 1780)
    *cat* V1*: move****_roll*** *V2: move_****downhill***
    cat move_**manner** move_**path**
    ‘The cat rolled down the hill.’

Senghas et al. (2013) later discovered a transitional state in LSN’s trajectory. They analyzed descriptions of manner and path events in child homesigners in Turkey, the signers who created LSN (i.e., LSN1), as well as the signers who learned LSN from Cohort 1 a decade later (i.e., LSN2). They found a “mixed” form, in which one verb conflates manner and path, combined with a second verb that represents either manner or path (but not both; see also Goldin-Meadow et al., 2015b). They first appear in homesign, peak in LSN1, and are rarely found in LSN2. (The criteria for assigning LSN signers to cohorts [LSN1 and LSN2] are described in detail in Section 2.4.)

Brentari et al. (2020) analyzed SVPs and MVPs in classifier predicates in four sign languages—ASL, BSL, HKSL, and Italian Sign Language (LIS)—to examine variation in the use of SVPs and MVPs expressing agency and number. They found that object handshapes and lateral movements did not always express non-agentive meanings; in contrast, handling handshapes and midsagittal movements were primarily restricted to agentive meanings.^5^ Object handshapes and lateral axis thus appear to be default forms, such that the distinction is [+agent] /ø agent (Pfau & Steinbach 2006).

Handshape type (handling) and movement axis (midsagittal) redundantly mark agency. However, as Pfau and Steinbach (2006) have noted, the midsagittal movement axis can be blocked when movement is repeated to indicate plurality. Figure 3 illustrates this point. All three forms in the figure are SVPs, repeated to express plurality. But the three forms express agency differently. In Figure 3a, both handshape (handling) and axis (midsagittal) redundantly express [+agent]. In Figure 3b, only handshape (handling) expresses [+agent], and axis (lateral) is a default form. In Figure 3c, only axis (midsagittal) expresses [+agent] and handshape (object) is a default form. Brentari et al. (2020) found that in BSL, HKSL and LIS, signers tend to produce single verb forms as in Figure 3a, combining repetition and midsagittal axis (no blocking); however, ASL signers tend to produce single verb forms as in Figure 3b, blocking the use of midsagittal with repetition. Verb forms like Figure 3c were much less frequent, suggesting that repetition blocks movement marking (midsagittal) more frequently than handshape marking (handling) for agency.

**Figure 4:**
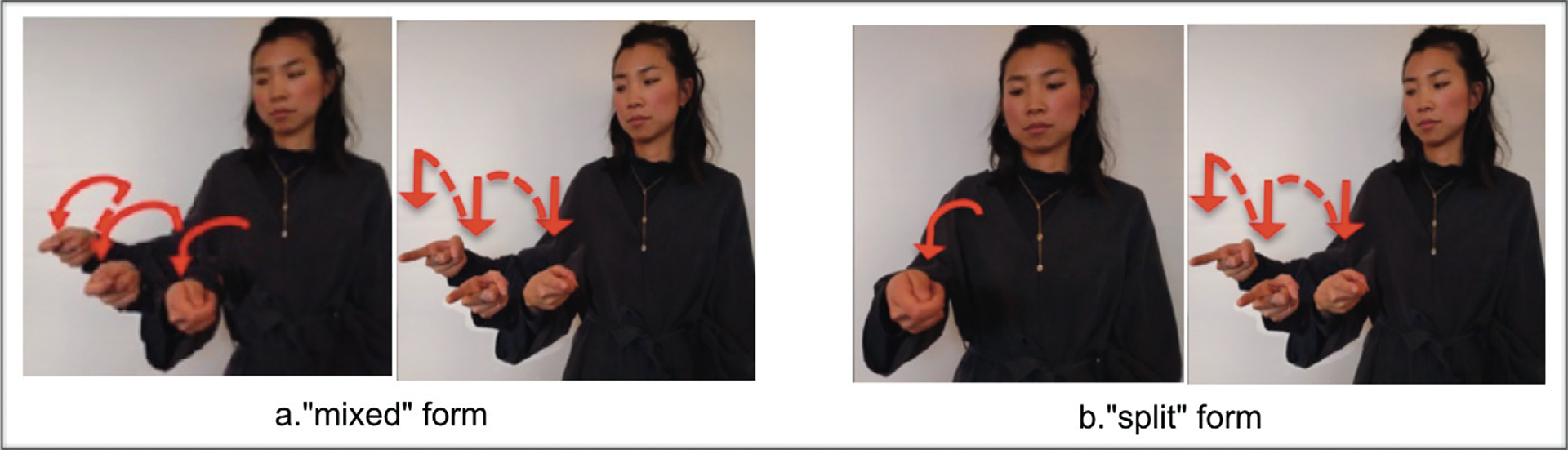
Multiple-verb predicates (MVPs) illustrating: (a) a “mixed” form with partial redundancy on V1 and V2: V1: **plural** (repetition) and **agent** (handshape and axis) and V2: **plural** (repetition) and no-agent (handshape and axis); (b) a “split” form with no redundancy between V1 and V2: V1: **agent** handshape (handshape and axis) and V2: **plural** (repetition) and no-agent (handshape and axis).

More relevant for the current study is redundancy across verbs in MVPs. Figure 4a illustrates an MVP that is partially redundant across V1 and V2: both V1 and V2 are marked with repetition for plural, but only V1 is marked for [+agent] (on both handshape and movement axis). We call these forms “mixed,” as in Senghas et al. (2013) and Goldin-Meadow et al. (2015b). Figure 4b illustrates an MVP that is not redundant: V1 is marked for agent (again on both handshape and movement axis) but has no marking for plural; V2 has repetition and is thus marked for [+plural] but has no marking for agent (it instead uses two default forms, object handshape and lateral movement). We call these forms “split.”

BSL, HKSL, and LIS signers’ MVPs containing [+agent]/[+plural] resemble those in Figure 4a; that is, “mixed” forms where one verb uses handshape (handling) and movement axis (midsagittal) to mark agent, and movement repetition to mark plural; the second verb *redundantly* marks plural using the default handshape and motion forms with movement repetition. In contrast, ASL signers’ MVPs containing [+agent]/[+plural] meanings resemble those in Figure 4b, that is, “split” forms where one verb uses handshape (handling) and movement axis (midsagittal) to mark agent (no repetition); the second verb *uniquely* marks plural using the default handshape and motion forms. Thus structures for agency and number appear to vary systematically across sign languages.

To summarize this section, we see that sign languages use simultaneous layering of meaning within words more than spoken languages do, as seen in Figure 1, and producing complex predicates as SVPs is very common in sign languages, more so than in spoken languages. However, not all complex predicates are SVPs, as we see in as we see in examples (5)–(8).

Since new languages are found only among sign languages, we analyze single-verb predicates (SVPs) and multiple-verb predicates (MVPs) in three groups of signers in Nicaragua—sign vs. sign+sign structures in classifier predicates. We analyze the emergence of form-meaning pairings to express agency (agent/no-agent) and number (singular/plural) in this new language. These data allow us to track the process of change over a shorter time frame, in a more granular manner, and at a different moment in historical time, than is typically possible in older languages.

### 2.3 The emergence of LSN

The creation of a new sign language in Nicaragua over the past five decades offers us the opportunity to observe the earliest stages of agent and plural structures as they emerge. Before the 1970s, deaf Nicaraguan individuals had little contact with each other (Kegl & Iwata 1989; Polich 2005; Senghas et al. 2005; Coppola 2006; 2020). There were periods when various classrooms and clinics were available to young children, but intergenerational contact and formation of a deaf community were hindered by the lack of a unifying national educational system, societal attitudes that isolated deaf individuals, and marital patterns that generally precluded hereditary deafness. One school, founded in Managua in 1974 with 25 deaf students, became publicly accessible in 1979 and expanded to include approximately 50 deaf students. In 1981, the school had a total enrollment of 500, with 125 deaf students, and by 1983, the school served 251 deaf students (Polich 2005). For the first time, a social community of deaf signers existed, with continuity from childhood through early adulthood. Today, LSN signers in Managua continue to socialize frequently at the National Nicaraguan Deaf Association and in each other’s homes.

In projects documenting LSN, researchers have divided signers into cohorts based on their year of entry into the signing community. The first cohort of signers began life as homesigners, the term used for deaf individuals who do not have access to spoken language and are not exposed to a sign language (Goldin-Meadow 2003). They were brought together as children, and they formed a deaf community in the late-1970s and early-1980s. Although the teachers at the school initially used spoken Spanish exclusively, these early signers produced and saw the signing of the other students during free periods outside of class. The resulting language is called the “initial contact variety,” and the signers are considered the first cohort of LSN signers (LSN1; Senghas et al. 2005). LSN1 signers have had the opportunity to sign with each other, but there was no pre-existing sign language model when they entered school. A second cohort of signers (referred to here as LSN2 signers) includes deaf individuals who entered the school in its second decade, from the mid-1980s to the early-1990s. In their early school years, they interacted with teenagers already at the school who were LSN1 signers. LSN2 and subsequent cohorts (LSN3, LSN4, etc.) developed a “sustained contact variety” (Senghas et al. 2005); in addition to a shared, deaf signing community, they have grown up with access to the signing produced by the previous cohorts as a language model.

The majority of deaf individuals in Nicaragua are not part of this signing community and do not know LSN. Due to a variety of social, geographic, and financial obstacles, they do not go to school or regularly interact with other deaf people. These deaf individuals are considered “homesigners”; see Coppola (2020) for a brief review. The homesigners included in this study have hearing losses significant enough to prevent the acquisition of a spoken language; they have not experienced regular exposure to LSN or to formal education; and none has successfully learned written or spoken Spanish. In addition, the homesigners do not interact with each other, do not have regular interactions with deaf or hearing signers of LSN, and have been using their individual homesign systems as their primary language for their entire lives (Coppola 2002). Each homesigner has a unique communication history with hearing family members and friends with whom they interact regularly (Coppola 2002). The hearing communication partners often engage with homesigners using signs and gestures, but the homesigners’ systems are not fully taken up by these hearing communication partners at either the lexical (Richie et al. 2014; Quam et al. 2022) or syntactic (Carrigan & Coppola 2017) levels.

The current study uses an apparent time approach (Labov 1994; Cukor-Avila & Bailey 2013), comparing different groups of signers in Nicaragua today to reconstruct the history of predicate constructions in LSN. Specifically, we examine whether morphemes are produced simultaneously on a single verb or are distributed across multiple sequential verbs in the predicate. We focus on meaning-form pairs associated with *agency* (no-agent/agent) and *number* (singular/plural). In cases where sequential forms are used, we also ask how the information is packaged, and the extent to which the information in an MVP is repeated in both verbs (and thus is redundant) or split between the two verbs (and thus is not redundant).

### 2.4 Research Questions

Because signers representing these early stages of language emergence continue to live and use their language today, we have the opportunity to reconstruct the emergence of specific structures within LSN by making comparisons in a controlled way across groups of present-day signers using the apparent time approach. Signers with different language experiences at different points in time and under different social circumstances reflect different historical time points in the development of LSN. In the current analysis, we ask whether there is a systematic *diachronic* pattern in how agency and number are expressed at three points in the emergence of LSN: Homesign, LSN1, LSN2. We ask two questions.

- How are agency and number expressed?
  - Are they expressed simultaneously in a single-verb predicate (SVP), or sequentially in a multiple-verb predicate (MVP)?
  - Does simultaneity/sequentiality vary across these three stages of language emergence?
- How are meanings packaged across the two verbs in an MVP?
  - Does one verb in an MVP express both agency and number?
  - Is the information expressed across the MVP partially redundant (“mixed”) or not redundant at all (“split”)?
  - Does this pattern vary across the three stages of language emergence?

## 3 Methods

We elicited descriptions of a set of scenes from all participants. These scenes showed objects with various motions and locations explicitly designed to encourage the production of sign language classifier constructions. Participant, stimulus, data, and analysis files can be found in the following OSF repository: https://osf.io/up8xv/?view_only=0b9b6568f1e44c2391dd9a3a75ecb334

### 3.1 Participants

Twelve Nicaraguans participated in this study: Four homesigners (ages 20, 24, 28, and 29), four first-cohort LSN signers (LSN1, ages 33, 34, 40, and 43; year of entry 1974–1982); and four second-cohort LSN signers (LSN2, ages 21, 25, 26, and 26; year of entry 1989–1994). All participants were deaf from at least early childhood, and the LSN signers all began signing by the age of 5, typically when they entered school.

### 3.2 Stimuli and Design

The stimulus items were drawn from a set of 64 scenes, 8 objects in 8 different scenes—photographs and short video vignettes, balanced for agent and no-agent items, and for items involving a single object or multiple objects. The objects in the stimulus clips exhibit a typical range of colors, shapes, and sizes. The eight objects are: toy airplanes, books, coins, lollipops, marbles, pens, television sets, and tweezers. The stimulus objects and vignettes were chosen according to their iconic affordances based on previous work on LSN and other sign languages, as discussed in Section 2.3—affordances for handling and object handshapes, and for single and repeated forms. Based on previous studies, we chose stimulus objects whose noun labels in LSN did not employ the same handshape as the handling or object classifiers typically associated with the objects, thus ensuring that we would be able to easily differentiate nouns and verbs in the responses. Each object was portrayed in 4 conditions (2 scenes per condition): a single stationary object without an agent (no-agent_singleOBJ), multiple stationary objects without an agent (no-agent_multipleOBJ), a single object acted on by an agent (agent_singleOBJ), and multiple objects acted on by an agent (agent_multipleOBJ). The study design is shown in Table 1. Excluding the table (the surface as a “ground” locative), which was present in every stimulus, each vignette offered the possibility to include one or two arguments in the description—the target object (conditions 1–4) or the target object and agent (conditions 5–8).

### 3.3 Procedure

Signers were instructed in their respective variant of LSN or homesign system to watch each vignette and describe what they saw to an interlocutor. The elicitation task is straightforward and does not require elaborate instructions. For this reason, and also to accommodate the homesigners, the instructions provided to all groups were minimal. The interlocutor was a familiar communication partner for the homesigners, and a peer from the same cohort for the LSN signers. Data collection sessions were videotaped and the video files containing the participants’ responses were annotated using ELAN (Crasborn & Sloetjes 2008).

### 3.4 ELAN Annotations

The descriptions produced by each participant were annotated by two of the authors (LH and DB). Each annotator completed half of the data annotations, and each also annotated 10% of the other’s items to serve as a reliability check. When disagreements occurred between annotators, the forms were discussed so that resolution could be achieved. The following properties were annotated for each description. It is important to note that we wanted to employ the same criteria for annotating all of the signers in all groups. A spreadsheet showing how each response was coded is available in the project OSF repository.

#### Intonational Phrases (IP):

The descriptions of LSN1 and LSN2 signers were considered single Intonational Phrases as long as there was no false start or self-correction. The homesigners were less fluid, and the communication partner tended to repeat the description produced by the homesigner to demonstrate understanding. To be sure we were comparing similar units across participants and groups, we used a long pause of over 500 milliseconds for establishing an IP. Intercoder agreement for Intonational Phrase breaks was 96%. Only the first IP of a description was used in order to be consistent across participants (and to make it more likely that we used homesigner responses that had not been influenced by their communication partners, cf. Goldin-Meadow et al. 2015a). There was 95% intercoder agreement on the location of the first IP boundary.

#### Label (noun) vs. Event (verb):

The descriptions were segmented into signs used to label the object (typically these were produced on the body or in neutral space without reference to a specific location) and signs used to describe the event or spatial arrangement shown in the vignette. The controlled task used in this study allowed this distinction to be made without difficulty. Inter-coder reliability for label (noun) vs. event (verb) was 100%.

#### Agency/handshape:

The *handshape types* were coded as *object handshapes (OHSs)*, *handling handshapes (HHSs)*, or *other handshapes*. As described earlier, handling handshapes are handshapes that iconically represent a hand holding an object. Object handshapes represent the entire shape of an object (sometimes referred to as entity classifiers) or one dimension of the object (referred to as size and shape specifiers, SASSs). As stated in the introduction, in many sign languages, including LSN, a handling handshape is considered a marker of an agent structure, and an object handshape is considered a marker of a no-agent structure. Other handshapes comprised less than 5% of the data and included those used to trace the outline of an entity or handshapes that were ambiguous. Inter-coder agreement for coding handshape type was 96%.

*Agency/Axis* was annotated as either midsagittal or lateral. Movements were coded as *midsagittal* if they originated or ended with the signer as a reference point (i.e., if the primary direction of movement was towards the signer’s body or away from the signer’s body). Movements were coded as *lateral* if they were articulated without reference to the body (i.e., if the primary direction of movement was vertical or horizontal without significant change in position relative to the signer’s body). As stated in the introduction, the midsagittal axis is considered a marker of agent structures (Horton et al. 2015), and the lateral axis is considered a marker of no-agent structures. If the signer used either a handling handshape or a midsagittal axis, we counted the lexical item as an agentive form; all other forms were considered non-agentive. Inter-coder agreement for coding movement type was 94%.

#### Plural/Repetition:

Movements were annotated as no-repetition (−rep) when produced as a single movement; and as repetition (+rep) when the primary trajectory of movement was repeated one or more times. Repetition is considered a plural marker. Intercoder reliability for repetition was 98%.

#### Number of verbs in the predicate (SVP or MVP):

**Figure 5:**
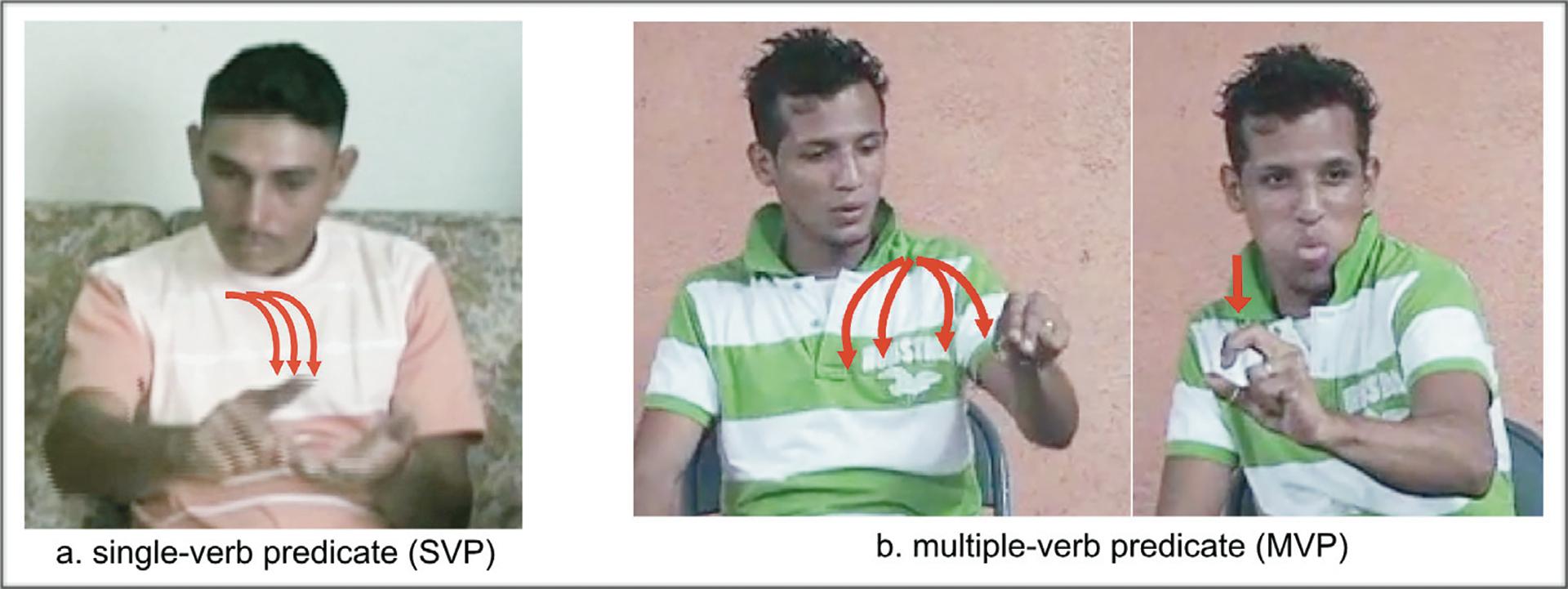
A sample single-verb predicate (SVP) and multiple-verb predicate (MVP) description from the data set for the same condition (condition 7): (a) illustrates an SVP with multiple midsagittal movements produced by a Homesigner for *putting multiple pens on a table in a row*; (b) illustrates an MVP produced by an LSN2 signer—V1 includes agent (handling handshape) + plural, and V2 includes no-agent (object handshape) + singular for *putting multiple marbles on a table in a row*. The videos of these responses, with glosses, are available in the project OSF repository.

Each distinct movement type was annotated—i.e., different verbal roots (move or be-located), or different direction or axes of movement—as well as the number of verbs in the predicate. An example of an SVP is provided in Figure 5a. An example of an MVP is provided in Figure 5b. Complete transcriptions for the descriptions in Figure 5 are given in (10)–(11).

A predicate was counted as an SVP if all of these criteria were met:

- there was one movement type (move or be-located) with a single direction or axis of movement.

Repetitions of that movement were counted as an SVP if:

- there were no intervening signs
- there was no pause between repeated movements of the same type greater than 300ms. For our purposes it did not matter if the repeated movements were punctuated by final holds (a period of stasis at the end of each repetition) or if they were produced in a smooth manner without final holds.

A predicate was an MVP if any one of the following criteria were met:

- there was more than one movement type (move or be-located) or different directions or axes of movement
- there were intervening signs
- there was a pause between repeated movements of the same type greater than 300ms.

- Figure 5a. Homesigner: Single-verb predicate (SVP) complete transcription
  *Six pen* V1*: move +* 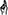 ***OHS_midsagittal axis+repeated***
  Six pen put_**long-thin-obj agent_plural**
  ‘[Someone] put pens (on a flat surface).’
- Figure 5b. LSN2 signer: Multiple-verb predicate (MVP) complete transcription
  *Table small Marble* V1:*move* + 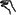 ***HHS+midsagittal axis+repeated***
  table small marble put_**small-round-obj_agent_plural**
  V2:*be-loc* + 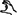 ***OHS+lateral axis***
  be-located**_large-round obj**
  ‘[Someone] put small marbles and one really large one on a table.’

## 4 Analyses

In this section, we analyze the distribution of SVPs and MVPs across participants, groups, and conditions; 630 descriptions were analyzed. We then focus on MVPs and ask how information about agency and number is distributed across the multiple verbs. Do the two verbs repeat the same information, or is the information split across the two verbs?

**Figure 6:**
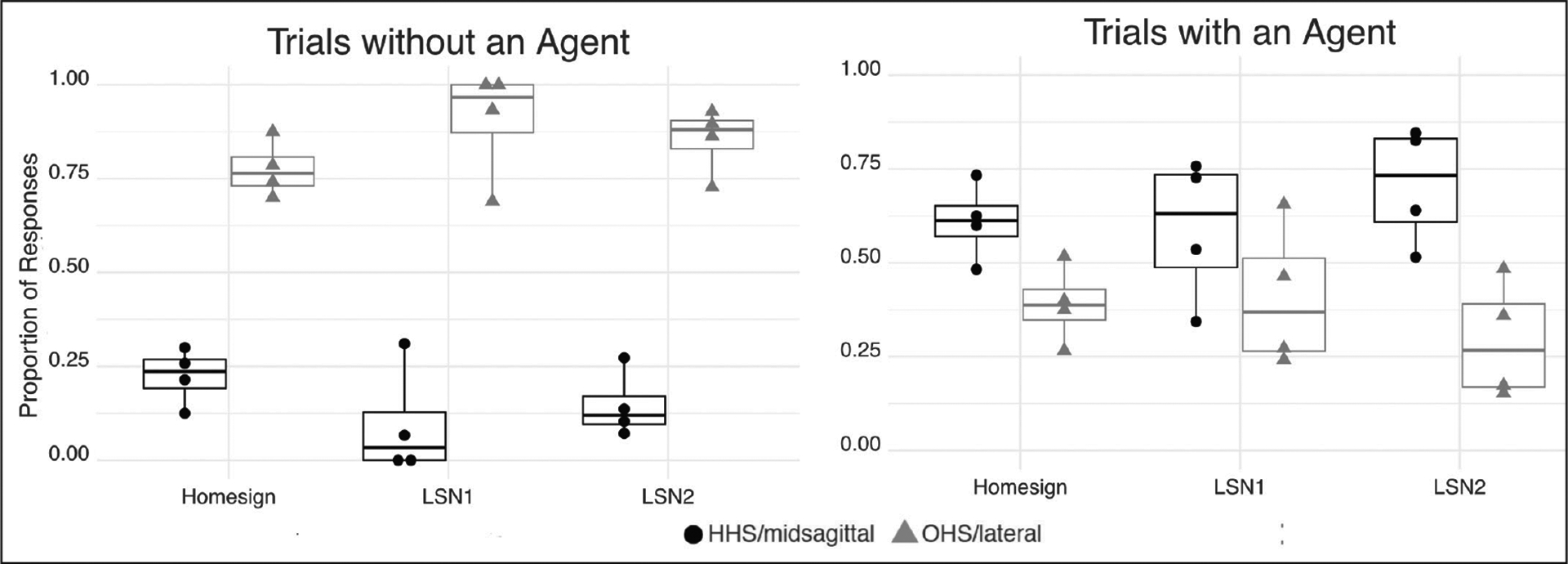
The proportion of predicates marked for agent for each of the three language groups (Homesign, LSN1, LSN2) for trials with (right) and without (left) an agent. **Agent** is marked by a handling handshape (HHS), midsagittal movement axis, or both (black dots). **No-agent** is marked by object handshape (OHS; gray triangles). Each dot represents a participant; the upper and lower bounds of the boxes indicate the first and third quartiles, and the horizontal bar in each box shows the group mean.

Our first step was to ensure that participants in each of the three groups used handshape and/or movement axis to mark agency, and repetition to mark number, as has been found in many sign languages (Brentari et al. 2015a; 2015b; 2020). Figure 6 presents the proportion of predicates marked for agent in agent and no-agent trials. A predicate was considered marked for agent if it contained a handling handshape, a midsagittal movement, or both; otherwise it was considered unmarked for agent.^6^ The three groups displayed the same pattern: Object handshapes and/or lateral movements were used more often than handling handshapes and/or midsagittal movements in no-agent trials; the handling handshape and/or midsagittal axis were used more often in agent trials. This pattern, found in all three groups of the current study, is consistent with the patterns found in previous studies.

**Figure 7:**
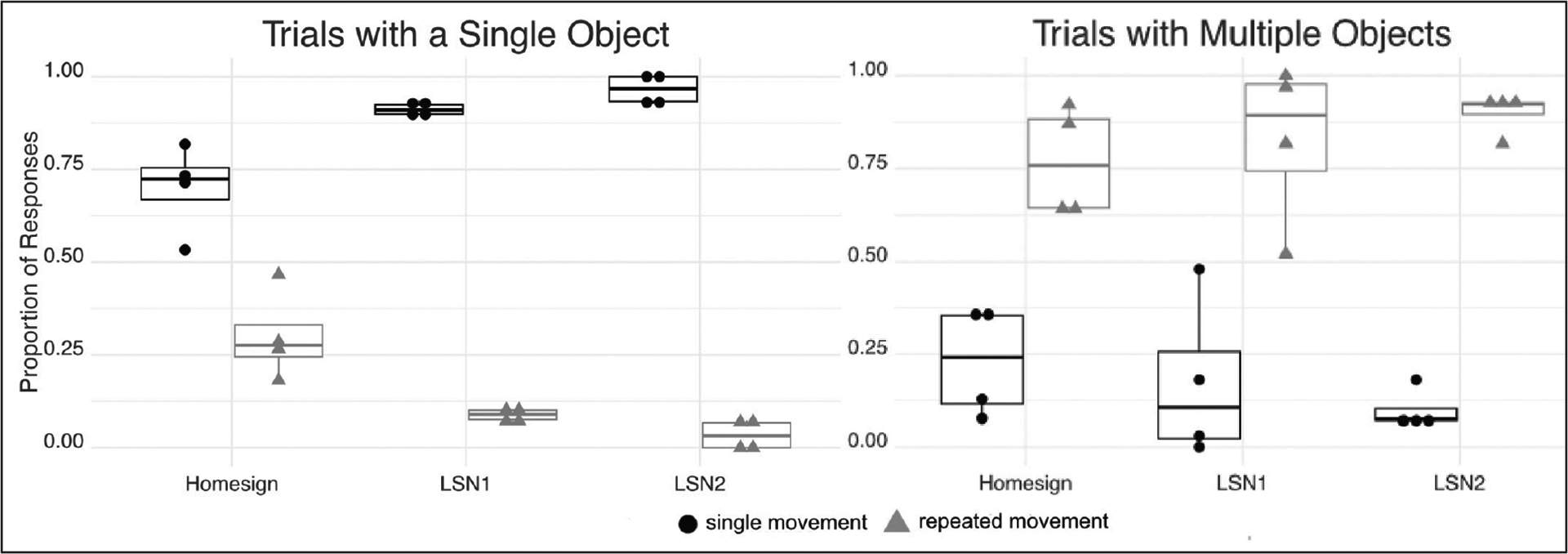
The proportion of predicates marked for **number** for trials with a single object (left) or multiple objects (right). **Singular** is marked by one movement (no repetition, black dots); **plural** is marked by repetition (gray triangles). Each dot represents a participant; the upper and lower bounds of the boxes indicate the first and third quartiles, and the horizontal bar in each box shows the group mean.

Figure 7 presents the proportion of predicates marked for number in trials with a single object or multiple objects. A predicate was considered marked for plural number if it was repeated one or more times. A predicate was considered unmarked for number if it was produced only once. The three groups displayed the same pattern: Single movements were used more often than repeated movements in trials with a single object; the opposite pattern was found in trials with multiple objects.

Results of a Spearman’s Ranked Comparison on the 445 data points provided by the LSN signers did not detect a significant relationship between their year of entry into the signing community and the proportion of expected forms combining agency and number marking: (*r*(6) = .59, *p* = .12).

### 4.1 Which factors predict the probability of participants producing single-verb or multiple-verb predicate responses?

We now turn to the general analysis of factors predicting the occurrence of SVPs and MVPs in the data. We fitted a mixed-effects logistic regression model to the data, using the “glmer” function, from the package *lme4* (version 1.1–27.1; Bates et al. 2015) in R (version 4.1.0; R Core Team 2021) to understand which stimulus conditions and participant groups are associated with the production of SVPs or MVPs, while accounting for nested and repeated measurements. 630 observations across participants and groups were analyzed. The random intercepts were Participant and Stimulus Object (8 object types shown in the stimulus vignettes). Model comparisons were performed using three models: M0, M1, and M2. We used M1 because it provided the best fit.

M0 is a baseline, unconditional model that included only the two random effects (Participant and Stimulus Object). M0 had an Intraclass Correlation Coefficient (ICC) of 0.32, indicating that the two random effects alone explained 32% of the variance in MVP responses.

M1 included three fixed effects in addition to the two random effects. The fixed effects were: Participant Group, with three levels: *Homesigners*, *LSN1* (reference group), and *LSN2*; Agency, with two levels: *no_agent, agent*; and Number (of objects) also with two levels: *singleOBJ* and *multipleOBJ*. Significance of categorical fixed effects was assessed using Wald’s ***χ***^2^ implemented in the car package (version 3.0–12; Fox & Weisberg 2019). Table 2 shows the regression table for M1. A third model, M2, included an interaction term between Agency and Number. The added term did not indicate a significant interaction effect (Wald ***χ***^2^ = 0.54, df = 1, p = 0.46), nor did it improve model performance using standard model comparison techniques (e.g., ΔAIC = 1.5, ΔBIC = 5.9).

In the model shown in Table 2 (M1), all of the fixed effects were significant, and the random effects accounted for 29% of the variance (see the ICC in Table 2), with relatively equal amounts of variance explained by Participant and Object (***τ*** values in Table 2). We observed the largest effect from the predictor Number (Wald ***χ***^2^ = 54.8, df = 1, p < 0.0001); MVP responses were produced more frequently in response to vignettes with multiple objects vs. vignettes with single objects. The model also indicated a large effect of Agency (Wald ***χ***^2^ = 5.11, df = 1, p = 0.02); MVPs were produced more frequently in response to vignettes with an agent vs. vignettes without an agent. Group also showed a significant but relatively weaker effect (Wald ***χ***^2^ = 8.9, df = 2, p = 0.01). There was a large confidence interval on the estimate (OR[95%CI] = 3.08[0.88–10.80], p = 0.078, Table 2). The difference between homesigners and LSN1 was not significant; however, LSN1 signers showed a trend toward fewer MVPs overall than either the homesigners or LSN2 signers.

**Figure 8:**
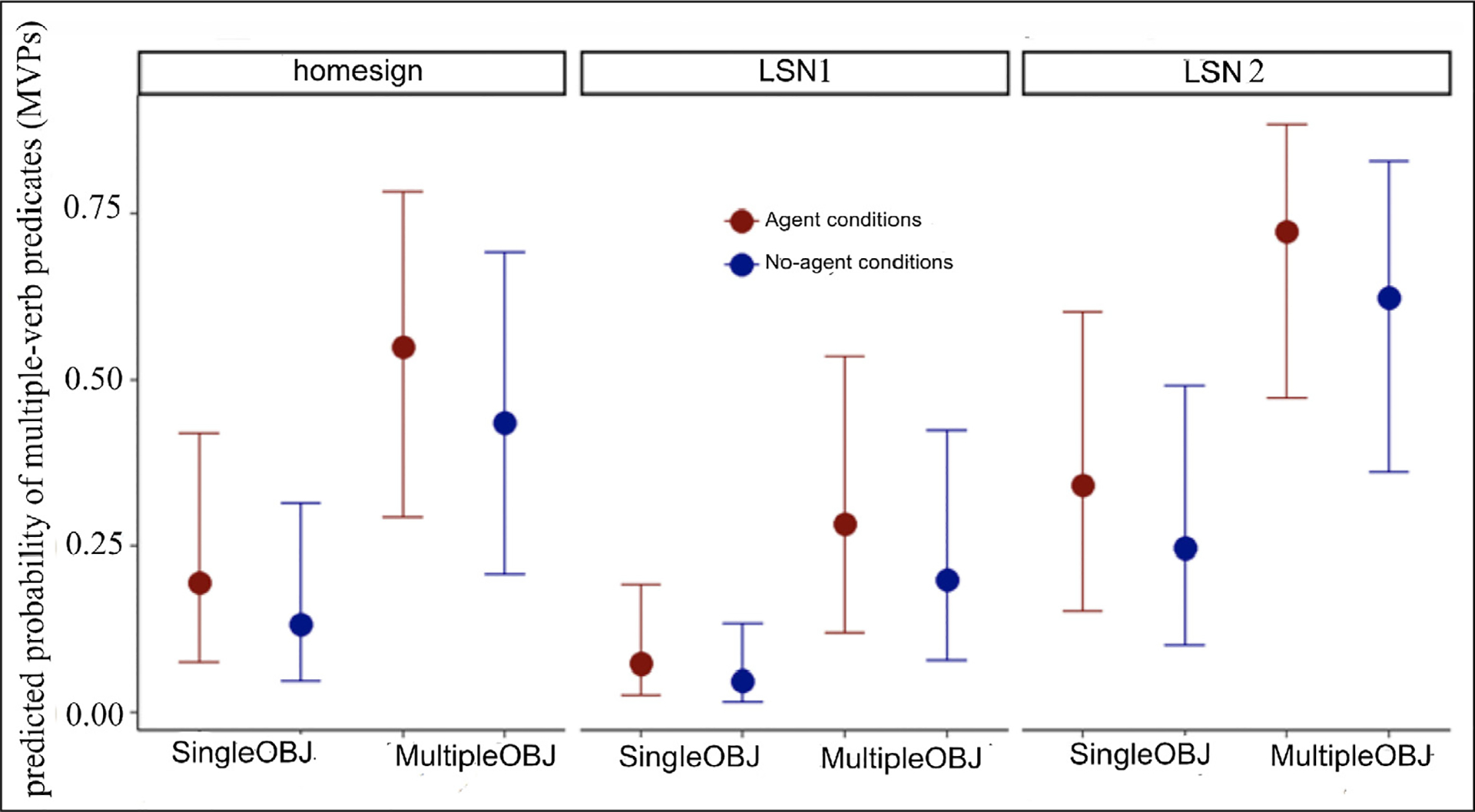
Predicted probabilities of a multiple-verb VP response in descriptions of scenes classified according to Agency (Agent, red circles; No-Agent, blue circles), and Number of objects (Single Object; Multiple Objects) in Homesigners, LSN1 signers, and LSN2 signers. The data set is available in the project OSF repository.

Figure 8 shows the predicted probabilities of an MVP response in descriptions of scenes classified according to Agency (Agent, No agent) and Number of objects (Single Object, Multiple Objects) in homesigners, LSN1 signers, and LSN2 signers for the best-performing model.

### 4.2 How are agency and number information packaged in MVPs?

To assess how agency and number information is packaged, we narrow our focus to descriptions of scenes that are designed to elicit both agent and plural markings (i.e., *agent_multipleOBJ* items). We address two questions in this section: Are agency and number produced simultaneously or sequentially? If two verbs are used to convey agency and number, is the information distributed across the two verbs, or is the information in the second verb redundant with the information conveyed in the first verb?

For each response, we first needed to identify whether agency and plurality information was included. Signers could mark both the agent and the plural, only the plural, only the agent, or neither. Table 3 shows that all groups produced [+agent] [+plural] marking more frequently than any other type of predicate description. When information is not included in the description, it is typically the agent, not the plural, that is omitted. Very few descriptions omit both an agent and plural form.

**Figure 9:**
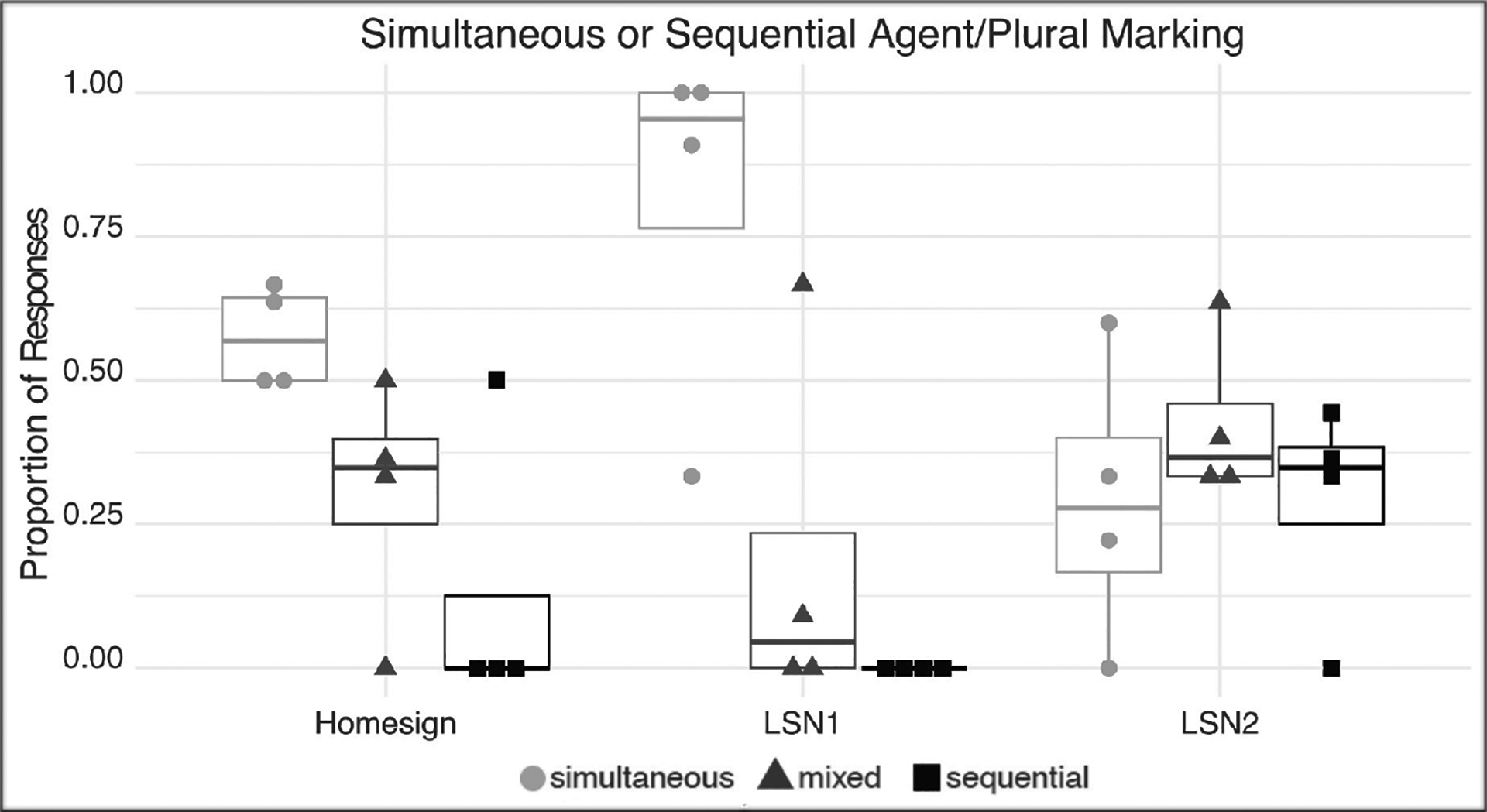
The proportion of responses marking both agent and plural classified according to whether the two are: simultaneously produced on the same verb (*simultaneous*: V1: agent+plural (gray dots)); the two are produced simultaneously on one verb, along with either marker on a separate verb (*mixed*: V1:agent+plural, V2: agent: (black triangles)); or the two are produced sequentially, agent on one verb and number on another (*sequential split*: V1: agent, V2: plural (black squares)). Each dot represents a participant; the upper and lower bounds of the boxes indicate the first and third quartiles, and the horizontal bar in each box shows the group mean.

To explore whether agent and plural marking are produced simultaneously or sequentially, we divided responses containing both agent and plural markings into three categories (85 data points). In *simultaneous agent+plural* SVPs, agent and plural are simultaneously produced on the same verb. In *sequential split agent–plural* MVPs, agent is produced on one verb and number is produced on the other. In *mixed* MVPs, agent and number are produced simultaneously on one verb, and either agent or number is produced separately on additional verbs. The results are presented in Figure 9.

The distribution of SVPs vs. MVPS differed across groups. A Mann-Whitney-U Test of Comparisons for small Ns) on SVPs revealed significant differences pairwise among the groups: homesigners vs. LSN1 signers, homesigners vs LSN2 signers, and LSN1 vs LSN2 signers all had significant differences (U = 1; p = .029)

**Figure 10:**
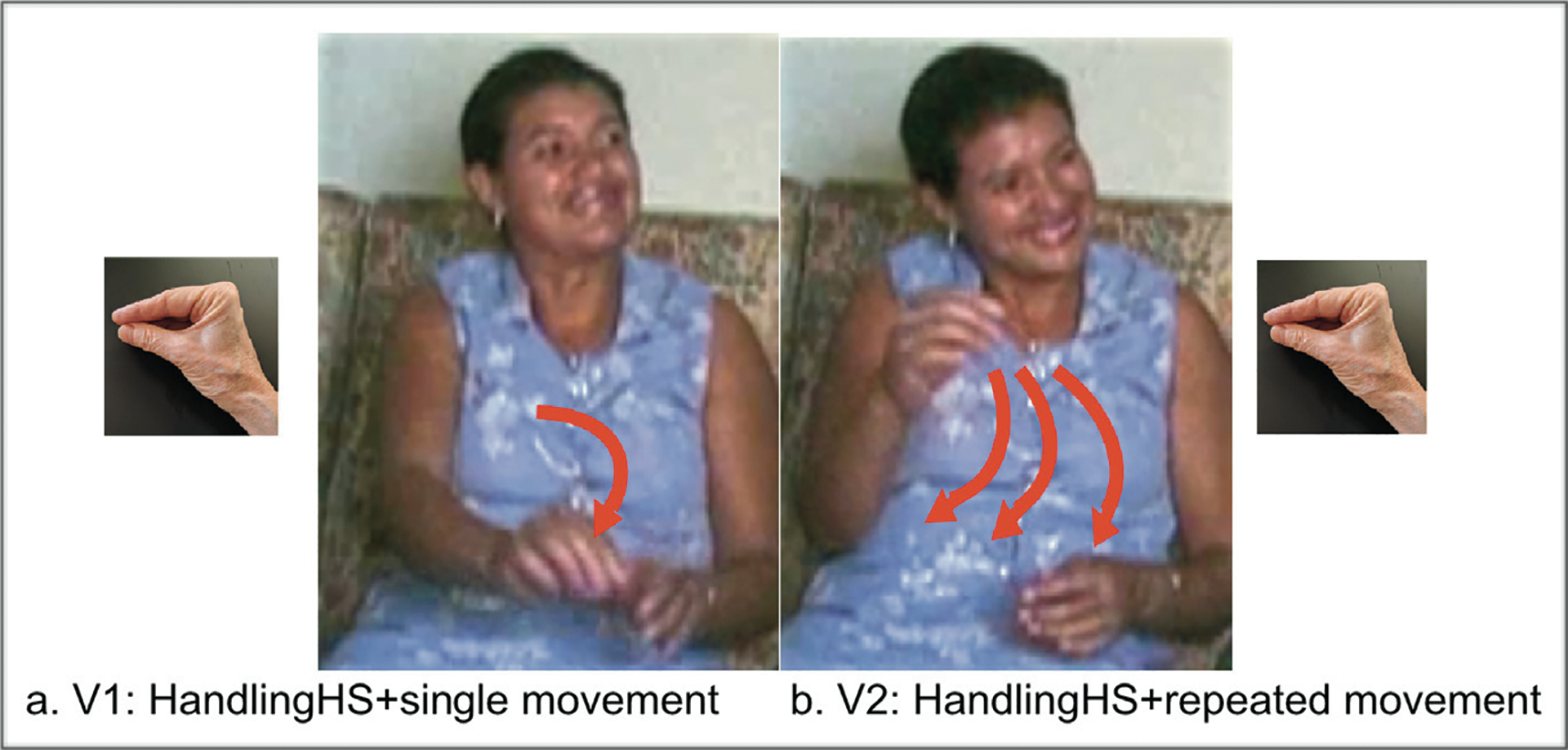
A homesigner producing a partially redundant *mixed* MVP to describe an agent *putting multiple planes on the table in a row* (condition 7).

**Figure 11:**
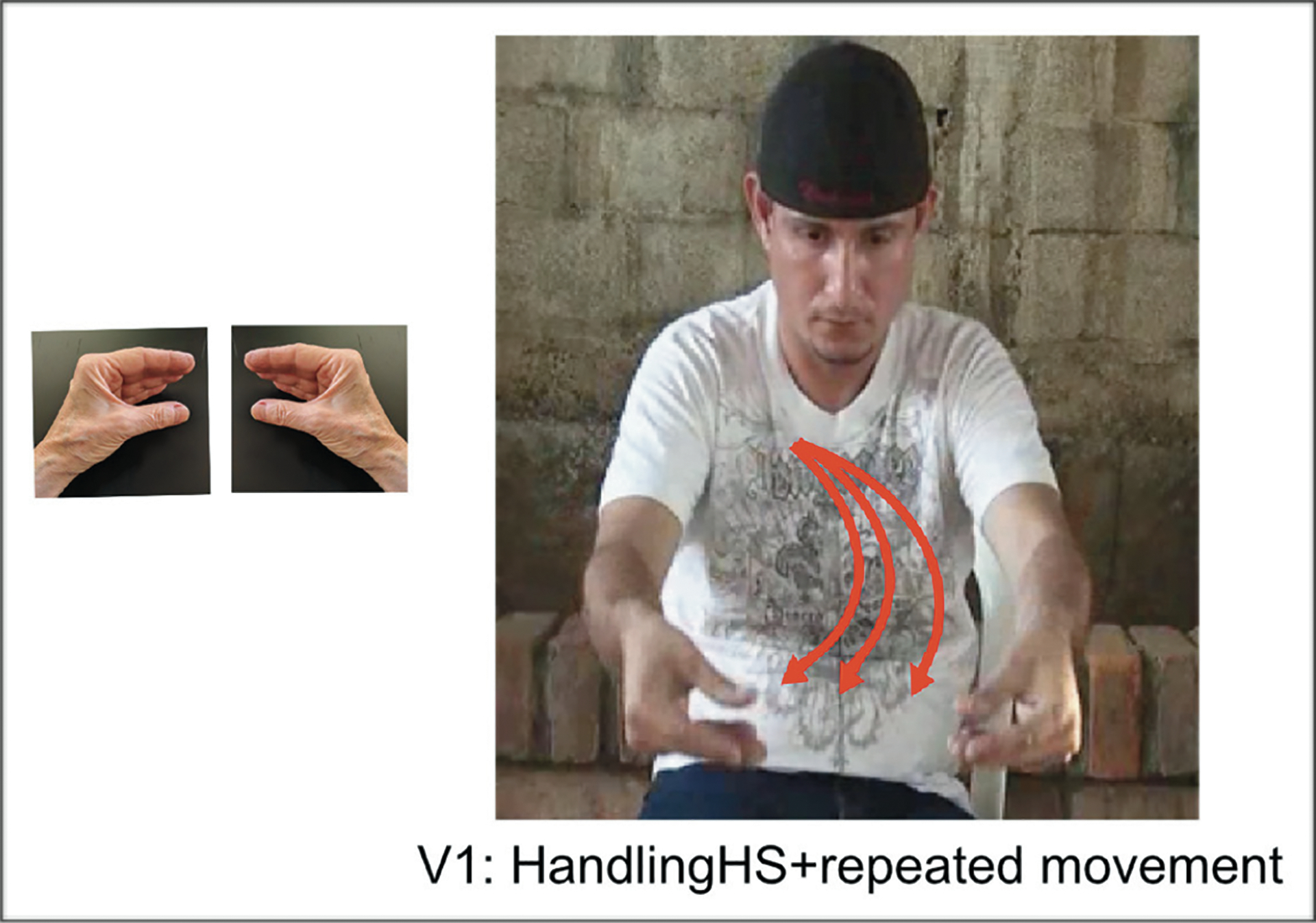
An LSN1 signer producing an SVP to describe an agent *putting multiple books on the table* (condition 7).

**Figure 12:**
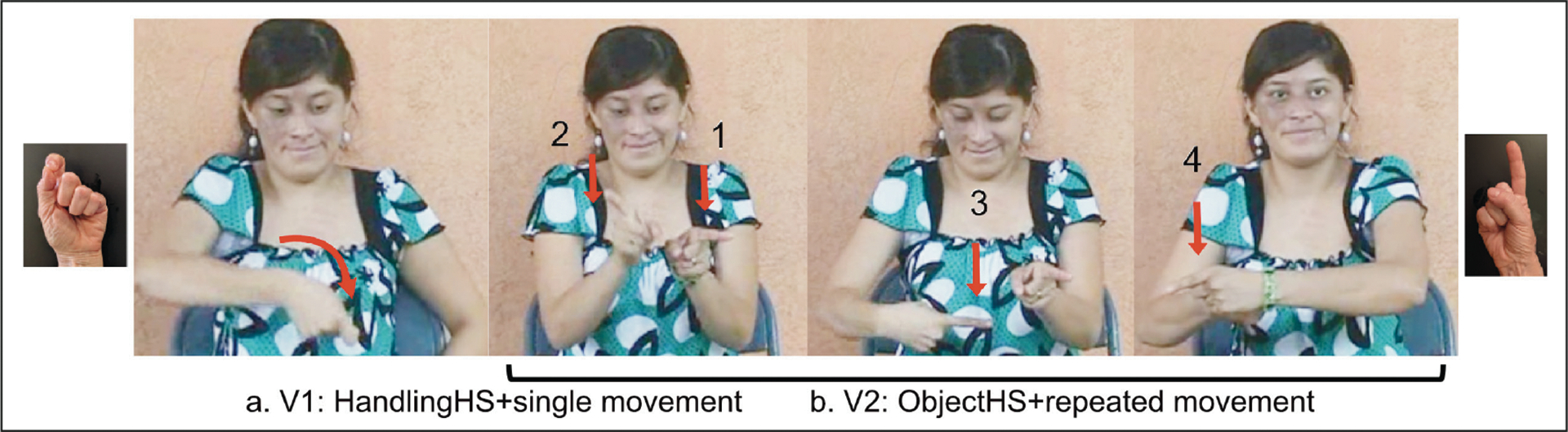
An LSN2 signer producing a *split*, non-redundant MVP to describe *an agent putting multiple lollipops on the table in a random arrangement* (condition 8).

Figures 10–12 provide illustrations of the [+agent] [+plural] response types (predicates only) shown in Figure 9. Complete transcriptions are given in (12)–(14).^7^ Figure 10 illustrates a “mixed” partially redundant MVP produced by a homesigner. The first verb is marked for agent (handling handshape + midsagittal axis) but not for plural; the second verb is marked for both agent (handling + midsagittal) and plural (repetition). Figure 11 illustrates an SVP produced by an LSN1 signer that is marked for both agent (handling + midsagittal) and plural (repetition). Figure 12 illustrates a sequential or “split” non-redundant form produced by an LSN2 signer. The first verb is marked for agent (handling + midsagittal) with a single, unrepeated movement (the default); the second verb is marked for plural (repetition), using the two default forms for no-agent (object + lateral).

- Figure 10. Homesigner: Multiple-verb predicate, mixed complete transcription
  *seven* V1*:move +* 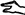 ***HHS+midsagittal axis***
  seven put_**small-obj_agent**
  V2*:move* + 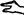 ***HHS+midsagittal axis+repeated***
  put_**small-obj_ agent_plural**
  ‘[Someone] put seven (on a flat surface).’
- Figure 11. LSN1 signer: Single-verb predicate (SVP) complete transcription
  *table book* V1*:move* + 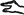 ***HHS (2 hands)+midsagittal axis+repeated***
  table book put_**thick-flat-obj_agent**
  ‘[Someone] put books on a table.’
- Figure 12. LSN2 signer: Multiple-verb predicate, sequential “split” (MVP) complete transcription
  *lollipop* V1*:move* + 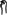 ***HHS+midsagittal axis***
  lollipop put_**thick-flat-obj_agent**
  V2*:be-located* + 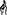 ***OHS (2 hands)+lateral axis+repeated***
  be-located**_long-thin-obj plural (random arrangement)**
  ‘[Someone] put lollipops (on flat surface) in a random arrangement.’

## 5 Discussion

Our findings suggest a number of generalizations about diachronic change in the frequency of SVPs and MVPs, and about how agent and number information is packaged in MVPs. Using an apparent time approach in which differences across groups of signers in Nicaragua can reveal diachronic changes in the emergence of LSN, we have shown that all three study groups use the structures and patterns of agent and pluractional marking that are used in other sign languages (Brentari et al. 2020). Object handshapes and the lateral axis of movement are more frequently used in non-agentive contexts, and handling handshapes and the midsagittal axis of movement are more frequently used in agent contexts; however, object handshapes and lateral axis of movement occur in both agent and non-agentive contexts, suggesting that they may be default forms in LSN, as they appear to be in other sign languages.

Looking first at SVPs vs. MVPs, we found that homesigners and LSN1 signers are more likely to produce SVPs than MVPs across all types of descriptions. Focusing only on descriptions that included both [+agent] and [+plural] marking, we found that LSN1 signers produced more SVPs than the other two groups. Looking next at how agency and number information is packaged in MVPs, we found that homesigners and LSN1 signers tend to produce forms that mark both agency and plurality simultaneously on the same verb; in contrast, LSN2 signers were equally likely to produce sequences with verbs that mark only one meaning, either agency or plurality—i.e., “split” forms. These findings echo earlier patterns found in the production of manner and path and verb agreement (Senghas et al. 1997; 2004; 2013), in which LSN2 signers frequently used a sequential, multiple-verb predicate for manner and path.

We have also shown that the signs of all three Nicaraguan groups use the structures and patterns of agency and plurality marking found in other sign languages (Brentari et al. 2020). First, object handshapes and the lateral axis of movement are more frequently used in non-agentive contexts; handling handshapes and the midsagittal axis of movement are more frequently used in agent contexts. Second, in [+agent][+plural] predicates, we see SVPs in LSN1 signers, forms that are common among BSL, HKSL, and LIS signers; and we see MVPs in LSN2 signers, forms that are common in ASL. Finally, we find *mixed* forms in all three Nicaraguan groups, particularly homesigners and LSN2 signers; these forms are common among BSL, HKSL, and LIS signers. We find sequential *split* forms in LSN2, and these forms are common in ASL. Thus, the forms observed across the stages of emergence in LSN replicate the variability found across sign languages around the world.

In this study, we did not analyze the degree to which the simultaneous expressions included componential structure. Some of the simultaneous forms may have been holistic; if so, the transition to sequenced forms would involve a reanalysis of the forms. Other simultaneous forms may have been composed of discrete meaningful elements; if so, the transition to sequenced forms would involve segmentation and recombination. In either case, our findings provide evidence that, in all groups, including homesigners, the elements of agency (handshape and axis) and number (repetition) are used in a componential way in the following sense: (i) agent and no-agent forms, and single and multiple forms, display distinct patterns of use that are consistent with how these forms are used productively in sign languages with longer histories (see Figures 2–4); and (ii) partially redundant “mixed” forms contain holistic forms alongside segmented forms, thus displaying the first steps of componentiality.

We now compare the MVPs that we have found in an emerging sign language with spoken language MVPs. The illustrations in Figures 11–12 can be situated within the spoken language literature and are parallel to Examples (15) and (16) below, from the language Watam, a language of Papua New Guinea (Foley 2008). In the redundant case, example (15), we see two verbs—one verb with the full form of negation affixed to it (*ba-…-tap* appears on *irik*; Eng., “go-down”), and a second verb with a partial form of the negative affixed to it (*ba-* appears on *uŋg*; Eng., “pull”). This pattern is similar to the “mixed” form displayed in Figure 4a and Figure 10.

- Partially redundant MVP negation forms in Watam, a Sepik-Ramu language of Papua New Guinea (Foley 2008:101)
  *yak kor* V1:***ba******-****uŋg-****ur*** V2: ***ba****-irik-****tap***
  1sg canoe **neg** -pull-**realis neg** -go.down- **neg**
  ‘I didn’t pull the canoe into the water’

In the non-redundant case, example (16), there is no negative form at all on *uŋg* (Eng., *pull*), but a full negative form appears on *irik* (Eng.: *go-down*). This pattern is similar to the split form in Figure 12.

- Symmetrical MVP: non-redundant negation in Watam (Foley 2008:101)
  *yak kor* V1*: uŋ g-****ur*** V2: ***ba****-irik-****tap***
  1sg canoe pull-**realis neg** -go.down- **neg**
  ‘I didn’t pull the canoe into the water’

Aikhenvald (2011) proposed that *lexicalization* is a common diachronic trajectory for both symmetrical and asymmetrical MVPs––more and more information is incorporated within a single verb over time. Contra Aikhenvald, the MVPs that we have observed in LSN are becoming increasingly segmented. On the surface, LSN appears to be following a trajectory opposite to the trajectory observed in spoken languages. One reason for the difference between signed and spoken languages could be modality. Spoken languages may typically start out sequential and gradually compress words to make them more simultaneous. Sign languages may initially favor simultaneous forms, and then, based on a number of factors, such as language experience, social factors, and historical time scale, users may divide at least some form-meaning pairings into sequential units (Aronoff et al. 2005; Senghas et al. 2013). A second possible explanation for the difference, as Aikhenvald (2018) mentions, is that different types of morphology may differ in how they distribute themselves across verbs. For example, Aikhenvald notes that, in spoken languages, person morphology is likely to appear redundantly on all of the component verbs in an MVP in all languages (see (17) below from Aikhenvald 2022); in contrast, negation varies from language to language and is redundantly marked in some languages, and selectively marked in others (for negation, see also Zeiljstra, 2022).

- Redundant person agreement on each component of the serial verb in Tariana (Aikhenvald 2022)
  ***du****ha Kumatharo* V1:***du****-mara-pida* V2:***du****-ka* V3:***du****-sita*
  **she** Kumatharo **3-sg_fem**-move downstream-pres.rep **3sg_fem** -arrive **3sg_fem** finish ‘She, Kumatharo, has arrived downstream.’

A third explanation, which we favor, is that our analyses of LSN were conducted at a different stage in the language’s diachronic development than were the analyses of indigenous languages of Americans studied by Aikhenvald and Muysken (2011); that is, the difference between LSN and spoken languages may stem from sampling data at different moments in the histories of the two types of languages. In Nicaragua, we are observing the very beginnings of a language, where new independent words, rich with meaning, are being pulled apart and serialized for the first time. In contrast, spoken language data come from languages that have been used for centuries, in which words have undergone lexicalization, phonology changes, and innovations in meaning. Change may be cyclical, moving from simultaneous forms to sequential forms (the changes we have described here), back to simultaneous forms (the changes described by Aikhenvald 2011). The steps we have captured in LSN may therefore represent a different moment in the cycle than the changes described in spoken languages.

## 6 Conclusion

We end by returning to the question of whether verb serialization has a universal, diachronic explanation (Lefebvre 1991; Amberber et al. 2010; Aikhenvald & Muysken 2011; Aikhenvald 2018). Our results point to a more subtle and varied path than one might have expected on the basis of either the spoken or sign language literature alone. The implications of this work are that diachronic changes towards simultaneity (lexicalization) or sequentiality (serialization) may be propelled by a variety of pressures, including modality (spoken vs. signed language), language experience, social factors, historical time scale, and type of morphology. The changes we have observed in the emergence of LSN suggest another pressure to add to this list––having a language model to learn from may be an essential ingredient in moving from a simultaneous to a sequential system for expressing agency and number meanings. This concurs with Newport’s 1988 claim that native-signing children learning ASL, who also have a proficient language model, show an ‘analytic’ tendency in their early production of ASL classifier forms. Crucially, the diachronic changes in forms for expressing agency and number that we have found in an emerging new sign language are echoed in the synchronic variations observed in the signed and spoken languages around the world today.

## Figures and Tables

**Table 1:** Study Design. Stimulus images and videos are available in the project OSF repository.

Conditions	Agent	#objects	Arrangement
1. [object] on table	no	single	n/a
2. [object] on table upside down	no	single	n/a
3. Multiple [objects] on table in in a regular arrangement	no	multiple	regular
4. Multiple [objects] on table – random arrangement	no	multiple	random
5. Put [object] on table	yes	single	n/a
6. Put [object] on table upside down	yes	single	n/a
7. Put multiple [objects] on table in regular arrangement	yes	multiple	regular
8. Put multiple [objects] on table (random arrangement)	yes	multiple	random

**Table 2:** Regression table for the mixed-effects logistic regression model including 3 fixed effects (Group, Agency, and Number) and 2 random effects (Participant and Object Type). The output and details of the model are available in the project OSF repository.

*Predictors*	Response		
	*Odds ratios*	*CI*	*p*
(Intercept)	0.05	0.02–0.15	**<0.001**
Group [homesign]	3.08	0.88–10.80	0.078
Group [LSN2]	6.62	1.91–23.01	**0.003**
Number [plural]	5.04	3.28–7.73	**<0.001**
Agency [agent]	1.59	1.06–2.37	**0.024**
**Random Effects**			
σ^2^	3.29		
τ_00 Participant_	0.67		
τ_00 Object_	0.66		
ICC	0.29		
N _Participant_	12		
N _Object_	8		
Observations	630		
Marginal R^2^/Conditional R^2^	0.223/0.446		

**Table 3:** Proportion of responses marked for agent and/or number in descriptions of vignettes featuring an agent acting on multiple objects.

	both [+ag], [+pl]	plural [+pl]	agent [+ag]	neither [–ag, –pl]
Homesign	.57 (N = 24)	.25 (N = 9)	.15 (N = 6)	.03 (N = 2)
LSN1	.41 (N = 27)	.39 (N = 25)	.13 (N = 8)	.07 (N = 4)
LSN2	.63 (N = 34)	.24 (N = 14)	.11 (N = 5)	.02 (N = 1)

## Data Availability

Data and related files are available at the following OSF repository: https://osf.io/up8xv/?view_only=0b9b6568f1e44c2391dd9a3a75ecb334 Participant demographics Stimulus images and videos De-identified coding of responses R code and annotations Video clips of example responses used in the manuscript

## Footnotes

1 Meir et al. (2017) has also investigated similar phenomena.

2 See Kimmelman (2022) for some exceptions to this generalization.

3 These handshapes can also be used to distinguish nouns from verbs in some sign languages (Tkachman & Sandler 2013).

4 Gesturers have been observed to use more object handshapes when naming objects than when expressing actions, and for other types of patterned iconicity (Janke & Marshall 2017; Padden et al. 2013; 2015).

5 In ASL agentive, transitive verbs that are not classifier constructions, such as give, advise, and inform, plurals can be combined with repetition to achieve spatio-temporal aspectual meanings, which can include midsagittal axis. e.g., *[verb] + to each* (Klima & Bellugi 1979; Wilbur et al. 1983).

6 A portion of these data were originally presented in Goldin-Meadow et al. 2015a for half of these objects (4 objects) which were analyzed for handshape only (not for axis); this graph adds data on four more objects and includes midsagittal and lateral movement as a possible marker for agency.

7 The stimuli and video clips for the full descriptions of Figures 9–11 are available in the project OSF repository.
